# Optical Tweezers Apparatus Based on a Cost-Effective IR Laser—Hardware and Software Description

**DOI:** 10.3390/s24020643

**Published:** 2024-01-19

**Authors:** Martin Burdík, Tomáš Kužela, Dušan Fojtů, Petr Elisek, Josef Hrnčiřík, Roman Jašek, Marek Ingr

**Affiliations:** 1 Department of Informatics and Artificial Intelligence, Faculty of Applied Informatics, Tomas Bata University in Zlín, Nad Stráněmi 4511, 760 05 Zlín, Czech Republic; burdik@utb.cz (M.B.); jasek@utb.cz (R.J.); 2 Department of Physics and Materials Engineering, Faculty of Technology, Tomas Bata University in Zlín, Nám. T. G. Masaryka 5555, 760 01 Zlín, Czech Republic; elisek@utb.cz (P.E.); hrncirik@utb.cz (J.H.); ingr@utb.cz (M.I.); 3 Department of Computer and Communication Systems, Faculty of Applied Informatics, Tomas Bata University in Zlín, Nad Stráněmi 4511, 760 05 Zlín, Czech Republic; fojtu@utb.cz

**Keywords:** optical tweezers, laser trapping, filtering, calibration, data acquisition

## Abstract

Optical tweezers (OT), or optical traps, are a device for manipulating microscopic objects through a focused laser beam. They are used in various fields of physical and biophysical chemistry to identify the interactions between individual molecules and measure single-molecule forces. In this work, we describe the development of a homemade optical tweezers device based on a cost-effective IR diode laser, the hardware, and, in particular, the software controlling it. It allows us to control the instrument, calibrate it, and record and process the measured data. It includes the user interface design, peripherals control, recording, A/D conversion of the detector signals, evaluation of the calibration constants, and visualization of the results. Particular stress is put on the signal filtration from noise, where several methods were tested. The calibration experiments indicate a good sensitivity of the instrument that is thus ready to be used for various single-molecule measurements.

## 1. Introduction

Optical tweezers (OT), also called optical traps, are a versatile tool for the manipulation of micro-objects sized in the range of micrometers to tens of nanometers, invented by Ashkin in 1970 [1]. They are based on the ability of a focused laser beam to attract the micro-objects toward the focal point, provided that their refractive index is higher than that of the surrounding environment. Using the acousto-optic deflectors or precisely positioned piezoelectrically driven movable mirrors deflecting the beam by extremely small angular steps, the position of the focus within the focal plane can be changed in the nanometer scale. Together with the position detection by the quadrant photodiode detector (QPD) or position-sensing detector (PSD), the position of the target particle can be measured with nanometer precision, allowing us to measure the tiniest forces acting on the particle, often produced by a single (bio)molecule [2,3]. Typical use of the optical tweezers apparatus is in the research of molecular motors, i.e., enzymes (ATPases) facilitating the active transport of membrane vesicles and other cargos in eukaryotic cells. Conjugating such an enzyme to polystyrene (PS) beads and trapping this bead by an optical trap, it is possible to determine the force produced by this molecule and the velocity of its motion along with a cytoskeletal fiber, the step length, and other properties [4]. Besides this use, optical tweezers were used to trap also other particles, nanoparticles, cells [5], and even objects inside the cells [6]. A considerable advantage of optical tweezers, especially when biomolecules are concerned, is the fact that the beam energy of the near-IR laser (wavelength of 800–1200 nm), typically used for this purpose, is not absorbed by the specimen, therefore, the temperature does not grow during the experiment.

In recent years, development of the software for controlling and calibrating OT was published. The method of the positional calibration of the particle’s trajectory using the microscope camera was published in the TweezPal toolbox in 2010 [7]. The 2014 toolbox [8] describes the geometrical optics to study the force acting on the captured bead in OT. The Tweezpy toolbox [9], published in 2021, reviewed the techniques for force calibration based on analysis of high-frequency camera footage of a particle captured in an OT, also using a PSD detector, and programmed in the Python language. Implementations of other calibration methods and noise reduction in the software can be found in previous publications [10,11,12,13,14,15]. A method for noise reduction by the detector signal transformation in Fourier space was introduced in 2011 [16].

In this work, we present the development of an integrated software package to control a homemade dual optical trap experimental apparatus and the subsequent data processing. The traps are formed by a near-IR laser beam of 808 nm produced by a cost-effective 3.2 W diode laser. The program works in several modes. The first mode (called pre-processing) is concerned with controlling the peripherals. It enables the calibration of the primary mirrors and the detector for the proper data acquisition from the sample. The pre-processing part also contains data acquisition from the PSD or QPD using the DAQ board card. The second mode of the program (called post-processing) is used for noise elimination from the data, saving the data, importing and evaluating measured data, their visualization, and mathematical analysis for OT calibrations. The developed program allows the user to move the two traps independently in the focal plane of the objective in two directions, record the PSD signal, detect the projection of the trapping-laser light reflected from the trapped object, and filter the signal from noise. Furthermore, the recorded signal can be visualized in several ways, statically and dynamically, and the calibration of the optical trap can be carried out, both regarding the force constant of the trap and the distance measured by PSD. The instrument is, thus, ready for experimental work in various research fields.

The article is divided into three main sections (contains Introduction). In Materials and Methods (Section 2), we introduce the polystyrene beads in water dispersion for this study, the experimental OT device forming the dual optical trap, noise elimination methods used for filtering acquired data, software parts of the developed program, and the description of OT calibrations indicating the parameters of the constructed device. In Results and Discussion (Section 3), we demonstrate the OT calibrations on a series of experiments with PS beads, the generated trap’s potential, and the position distribution of the studied particle inside the trap. The following part shows the influence of filtration methods on measured experimental data and the comparison of our results with other similar studies.

## 2. Materials and Methods

Polystyrene beads (Sigma Aldrich, St. Louis, MO, USA, Latex beads, polystyrene LB30 and LB8) of diameter of 3 µm and 0.8 µm were used in this study. The prepared dispersion of 0.1 µL PS beads in 1 mL ultrapure water was loaded on the microscope slide and covered by the coverslip (VWR No. 1 20 × 20 mm). The edges of the coverslip were overlaid by oil to eliminate evaporation and flow of the dispersion. Due to the low concentration of presented beads, only one could be trapped in OT.

### 2.1. Experimental Device

**Figure 1:**
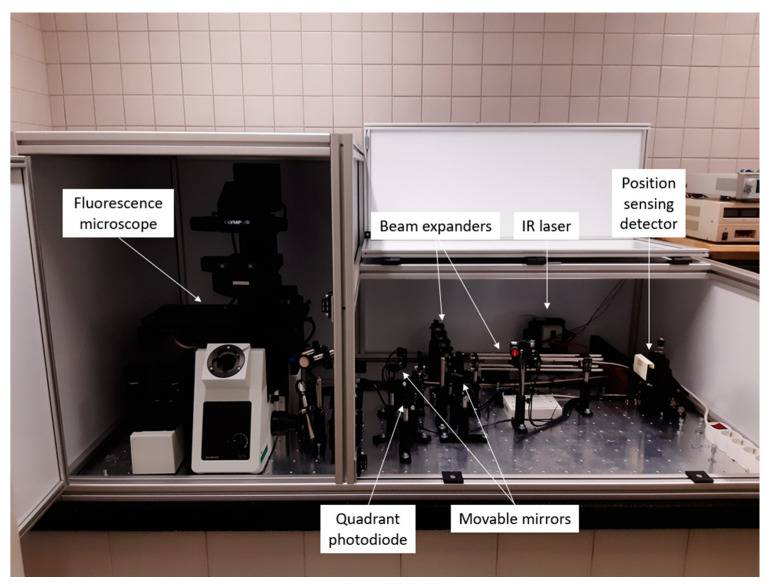
Apparatus for the optical tweezers experiments based on fluorescence microscope and IR laser on an antivibration platform covered by a protection box. The device is composed of optical elements, such as beam expanders and movable mirrors. The laser beam is detected using two detectors fulfilling different purposes.

A device for the formation of dual optical tweezers was constructed in our laboratory on the base of the Olympus IX-73 fluorescent microscope with the oil immersion phase objective 100× with NA 1.40 (UplanSApo, Tokyo, Japan) (Figure 1).

**Figure 2:**
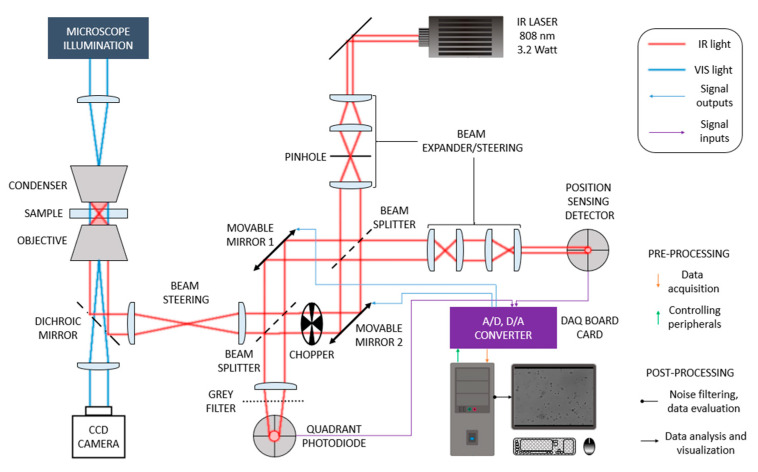
A scheme of the optical line generating the optical trap, adapted from [17]. The scheme also demonstrates electronically controlled parts and the flow of acquired signals.

A beam of a 3200 mW IR diode laser of the 808 nm wavelength bought from ECLIPSERA, s.r.o (laser-shop.cz) was focused on the observed specimen by a special optical line. The beam is split in two half-beams, independently deflected by the piezoelectrically controlled mirrors in two perpendicular directions, after which they are merged in the same line again (Figure 2).

Therefore, two optical traps can be formed and independently driven in the focal plane. The device is equipped with a PSD (Thorlabs, PDP90A) [18] allowing us to track the trapped particles precisely.

The position of the bead is detected by a PSD that detects the light of the trapping laser reflected by the trapped object. The reflected light is collected by the objective and lead along the same line as the primary trapping beam. After running through the two beam splitters, it runs through a projector that projects the focal plane of the objective on the plane of the PSD sensor. The projection of the sub-micrometer microscopic beads is of millimeter size. During the position detection, only one line can be opened, otherwise, a mixture of two signals would be detected. Therefore, if only one line is used, the other must be blocked. If, however, both lines trap experimental objects, the one in which the position of the trapped object is not measured is interrupted by a chopper and the signal (from the measured line) is detected only when this line is blocked. Although it is not frequently needed, signal detection from the chopped line may be facilitated by the subtraction of the total signal (chopper open) and the single-line signal (chopper closed). In this system, the position of the trapped object is measured relatively with respect to the center of the trap, no matter where the trap is. The position of the trap itself is known from the microscope image but is also detected, with higher precision, by QPD (Thorlabs, PDQ80A) [19] recording the position of the waste portion of the primary beam after passing the second beam splitter.

All data from the detectors are recorded by PC after the A/D conversion carried out by the DAQ board card (OMEGA Engineering, OMB-DAQBOARD-3000 Series card) [20]. Controlling the device and data processing are carried out by the program described in the following sections.

### 2.2. Noise Elimination Methods

#### 2.2.1. Causality of Filter

The non-causal filter is a filter where output depends on present, past, and future inputs. Non-causal filters can be implemented only in the case of offline signal processing.

The causal filter is a filter where output depends on present and past inputs. This filter can be implemented in both online and offline signal processing.

#### 2.2.2. Moving Average Filter

The moving average filter [14,15] is a useful and simple method for reducing noise. The principle of the data moving averaging in the causal variant is demonstrated by filter response $y\left(n\right)$ for n step

(1) $$y\left(n\right)=\sum_{k=0}^{M}\frac{1}{M+1}x\left(n-k\right),$$

where M is the order of the filter, and $x\left(n\right)$ is the n-th raw data value.

#### 2.2.3. Blocking Average

Another way of noise reduction is blocking average. The average of all values from the block replaces each block. Blocking reduces noise by compression from total number of raw data N to the number of data points after blocking M. The $y\left(n\right)$ of blocking average presents

(2) $$y\left(n\right)=\frac{M}{N}\sum_{k\in \frac{N}{M}}^{\;}x\left(k\right),$$

#### 2.2.4. Finite Impulse Response

Finite impulse response (FIR) filters [21] are generalizations of moving average filters by

(3) $$y(n)=b_{0}x(n)+b_{1}x(n-1)+\ldots +b_{M}x(n-M)=\sum_{k=0}^{M}b_{k}x\left(n-k\right),\;$$

(4) $$b=\left[b_{0},b_{1},\ldots ,b_{M}\right],$$

where $b_{0}\ldots b_{M}$ are vectors of filter coefficients called impulse response, and *M* is the filter order. Filter constant b can be obtained by inverse Fourier transform of the frequency response.

**Figure 3:**
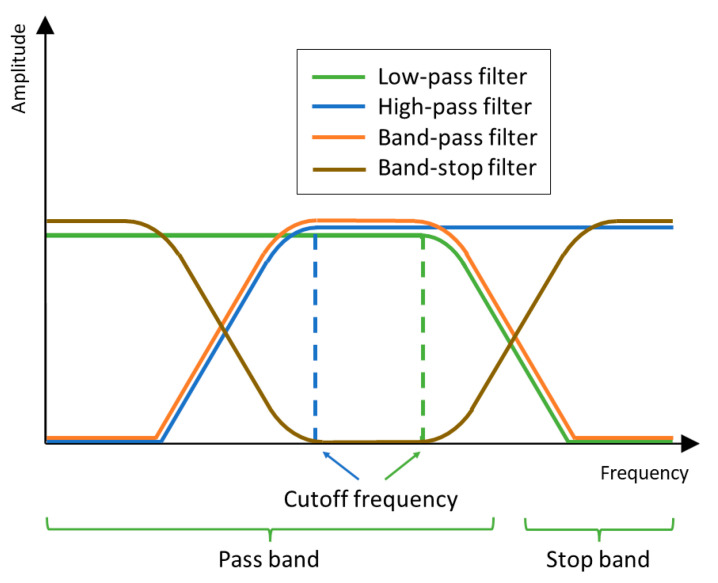
Frequency response of the low-pass (green line), high-pass (blue line), band-pass (orange line), and band-stop (brown line) filter.

Based on filter parameters (vector b), we can create four types of FIR filters (Figure 3). A low-pass filter is a filter that passes signals with a frequency lower than the cutoff frequency and attenuates signals with frequencies higher than the cutoff frequency. A high-pass filter is a filter that passes signals with a frequency higher than the cutoff frequency and attenuates signals with frequencies lower than the cutoff frequency. The band-pass filter is a filter that passes signals with a frequency in a given range of frequencies (lower cutoff and higher cutoff) and attenuates signals with frequencies out of the interval frequency. A band-stop filter is a filter that attenuates signals with a frequency in a given range of frequency (lower cutoff and higher cutoff) and passes signals with frequencies out of the interval frequency. In our case, the frequency response of the ideal low-pass filter can be used to remove noise from real-time PSD signal measuring of the bead’s trajectory in OT. A moving average filter, which was primarily used in this study, is a particular type of low-pass filter.

Implementation of a low-pass filter provides impulse response from frequency response. It can be performed with a method provided by Python’s SciPy library [22,23]. 

#### 2.2.5. Infinite Impulse Response

Finite impulse response filters depend only on previous, present, and future (in non-causal variant) inputs, while infinite impulse response (IIR) filters depend in prior outputs. The difference equation of the IIR filter is [24]

(5) $$y\left(n\right)=\sum_{k=0}^{M}b_{k}x\left(n-k\right)-\sum_{l=1}^{N}a_{l}y\left(n-l\right),$$

where $a_{l}$ and $b_{k}$ are the filter’s denominator and numerator polynomial coefficients. The main IIR filter advantage is high performance with very effective computationally.

### 2.3. Software Parts and Their Description

#### 2.3.1. Pre-Processing Mode

- Controlling the peripherals

For the correct operation of the optical trap, it is necessary to adjust the primary mirrors so that the incident laser beam is focused in the desired path. Peripherals in our apparatus are primary mirrors, chopper for defined laser beam cutting, laser source (TTL signal), and detectors.

**Figure 4:**
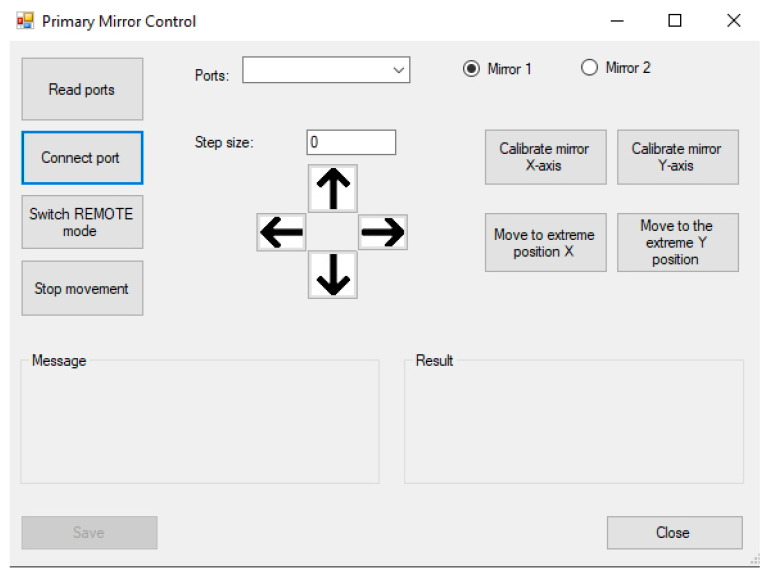
User interface of primary mirror control form. Left buttons connect the appropriate ports of DAQ board card. Central part is prepared for moving the traps individually by selecting and controlling mirror 1 or 2. The right part contains the functions to calibrate the mirror’s home position (or go to limit position) based on the detection of the waste signal of the trapping laser using QPD detector.

The position of the dual optical trap can be set using the *Primary mirror control form* (Figure 4) reading the available COM ports. The appropriate COM port in the DAQ board card to which the primary mirrors are connected should be chosen.

The selected port can be connected with the *Connect port* button and switched to the remote control with the *Switch REMOTE mode* button. The primary mirrors can then be calibrated by selecting mirror 1 (first optical trap) or mirror 2 (second optical trap) and pressing the *Calibrate X-axis* and *Calibrate Y-axis* buttons sequentially to reset the primary mirrors (prime laser beam) to the QPD detector’s center (zero voltage in vertical and horizontal axis). This helps us find the generated trap’s position in the microscope sample. Fine-tuning can be achieved by setting the *Step size* and pressing the arrow buttons according to direction where the primary mirrors move.

**Figure 5:**
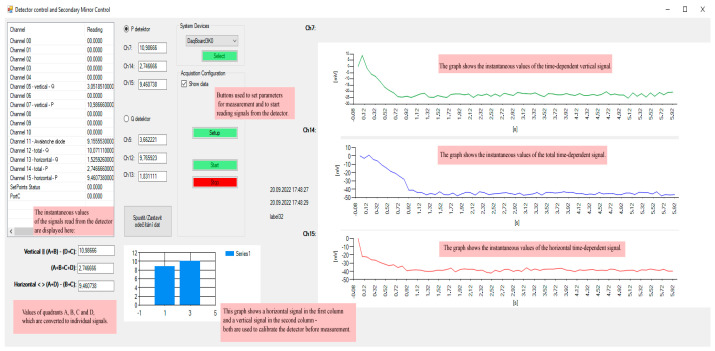
Detector control form—The pink boxes describe each software function and are not a part of the user environment. The left white window shows the active DAQ channels. Under that is the real-time value of X, Y, and aggregated signals. Three graphs in the right part are real-time rewriting signals by a defined time window used to calibrate the detector’s position. It is possible to select PSD (P detector) or QPD (Q detector).

The next necessary step is the detector’s calibration. After displaying the *Detector control form* (Figure 5), the tab of the respective detector should be selected—*System device*, which is determined using the *Select button*. Signals from the detector are read—horizontal (CH15), vertical (CH7), and aggregated (total) signal (CH14), according to which it is possible to evaluate in which direction the monitored object is moving.

The Show data checkbox should be checked in the Acquisition configuration, set the *Number of Scans* and *Scan Frequency*, and press the *Setup* and *Start* buttons. After pressing the *Start button*, a library created in Python starts, which provides the actual reading of data from the detector in real time.

The current values of the horizontal and vertical direction of the incident beam on the detector are displayed in the lower part. The micrometric screws of its holder should adjust the detector’s position so that the measured quantities of a blank sample are equal to zero, which means that the beam directly strikes the center of the detector.

The data from the detector are processed by the DAQ board card, which enables data acquisition from 16 analog input channels, 2 analog output channels, and 24 digital I/O channels. A total of 6 analog inputs and 2 analog outputs are used for the needs of data collection from the optical trap.

In the right part, the currently read data from the detector are presented in the vertical direction—the sum of two upper quadrants minus two lower quadrants (1st chart); in the horizontal direction—the sum of two left quadrants minus two right quadrants (3rd chart); and also the aggregated signal—the sum of all the quadrants (2nd chart). The left part shows the values of the current scanned data, which are displayed in the charts in the right part. It is also possible to save data in this form.

- Data Acquisition

Physical parameters are sampled into signals by the 2D lateral effect position sensor, which scans the radiation region from 320 to 1100 nm, or quadrant detector sensor head in the radiation region from 400 to 1050 nm.

Data acquisition can be made with the following steps [25]:

(1) Configuring channels;
(2) Configuring acquisition events;
(3) Setting the acquisition rate;
(4) Setting up the buffer model;
(5) Arming the acquisition;
(6) Triggering the acquisition;
(7) Monitoring the acquisition and receiving data.

(1) Configuring channels

Each acquisition needs to have a given sequence of channels scanned periodically. Our setup has three channels to acquire (vertical, horizontal, and aggregated). A basic configuration, gains, and flags for each channel are also set up. Gains g are amplifiers of a measured signal with a value from {1,2,4,8} that can adjust the amplitude power of the incoming signal. The acquired signal is in voltage, with a max value $V_{max}$ of used DAQ board card of ±10 V. It is possible to set the flag parameters for each scanned channel if the range is unipolar $r_{unipolar}$ or bipolar $r_{bipolar}$ [26]

(6) $$r_{unipolar}=\left(0,\;\frac{V_{max}}{g}\right),$$

(7) $$r_{bipolar}=\left(-\frac{\;V_{max}}{2g},\;\frac{V_{max}}{2g}\right),$$

(8) $$b_{res}=\frac{V_{max}}{g\times 2^{b_{depth}}},$$

where $b_{res}$ is a resolution of one bit and $b_{depth}$ of our system is 16.

(2) Configuring acquisition events

For each acquisition, events were defined to start and stop acquisition with defined scanning frequency. A start event can be as simple as starting the acquisition immediately upon arming and stopping in optional time (manual stop mode), or by setting the scanning frequency and number of scans (number of scans mode) to acquire the defined number of scans. This mode is helpful for every same length measurement, allowing comparable results.

(3) Setting the acquisition rate

**Figure 6:**
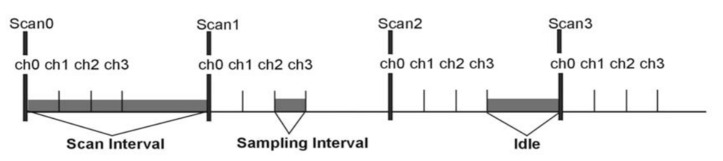
Acquisition rate, adapted from [14]. Between two scans (scan interval) a series of channels could be acquired (sampling interval).

The maximum sampling rate of the used device (DAQ board card) is 1 MHz divided by the number of acquiring channels. A scan interval is an interval where all channels are scanned once (Figure 6). The sampling interval is given by a DAQ and less than one µs. Thus, it is possible to acquire data from a single channel with a frequency of 1 MHz. For two channel data acquisition, the maximum sampling frequency is 500 kHz.

(4) Setting up the buffer model

Buffer models exist in two basic models: the user buffer model and the driver buffer model. In the case of the user buffer model, the user or application can allocate the buffer and pointers and handle them by themselves. The user or application is responsible for buffer maintenance. Linear or circular buffers can be used with the user buffer model. The driver buffer model takes responsibility for buffer management from user/application to driver.

**Figure 7:**
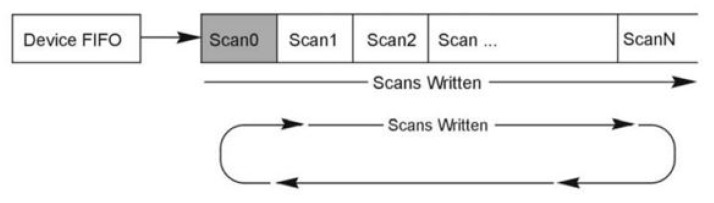
Buffer model, adapted from [14] demonstrating linear scans written (linear buffer) and written scans circularly (circular buffer).

- Linear buffer (Figure 7—straight scans written) is filled from the beginning of the buffer with the newest scans. Once the buffer is filled to the buffer capacity, the driver will stop writing new scans to the buffer even though the acquisition may continue. New scans are written into device FIFO if acquisition continues until FIFO overruns.
- Circular buffer (Figure 7—circular scans written) can never be overrun on level device FIFO. Once the buffer is filled to the buffer capacity, the new scans are written to the beginning of the buffer.

(5) Arming the acquisition

The acquisition can start when all configurations are achieved and the buffer is correctly set up. It means that acquisition is turned from the “IDLE” state to the “ACTIVE” state and is waiting for the trigger events.

(6) Triggering the acquisition

A trigger event can be one of the compatible trigger events [25].

(7) Monitoring the acquisition and receiving data

When a trigger event triggers the acquisition, it is possible to monitor the progress of acquisition and process data. This step returns the state of acquisition where the parameters are positioned in the buffer and where the next scan will be written.

#### 2.3.2. Post-Processing Mode

- Signal filtering

A discrete-time system is a computational algorithm for transforming a discrete-time input signal into an output. A digital filter is a mathematical algorithm that operates on a digital dataset, helps extract information of interest, and removes unwanted data, such as noise. For tracking the particle’s motion trajectory, it is necessary to proceed with a discrete-time system with a high-frequency signal with frequencies of 10 kHz or higher and eliminate the external noise from the background.

For this purpose, many variants of mathematical filtering methods exist that eliminate the external noise from measured data [27] (described in Section 2.2) while maintaining every trajectory (low frequency) detail.

The trajectory was measured by means of the two signals from PSD indicating the position of the trapped bead in X and Y directions. These signals were acquired with the frequency of 100 kHz. Such a high frequency allows us to carry out block averaging of the signals by 10-point windows in order to reduce the amount of data as well as the noise of the electronics. Typical acquisition time was 100 s, which is sufficient for representation of the low-frequency components of the signal that are necessary for the subsequent determination of the force constant of the optical trap. The noise of the signals was further eliminated using the moving-average filter. The determination of the optimum averaging window that removes majority of the noise but keeps the shape of the trajectory is described in Section 2.5.2.

- Data visualizations

**Figure 8:**
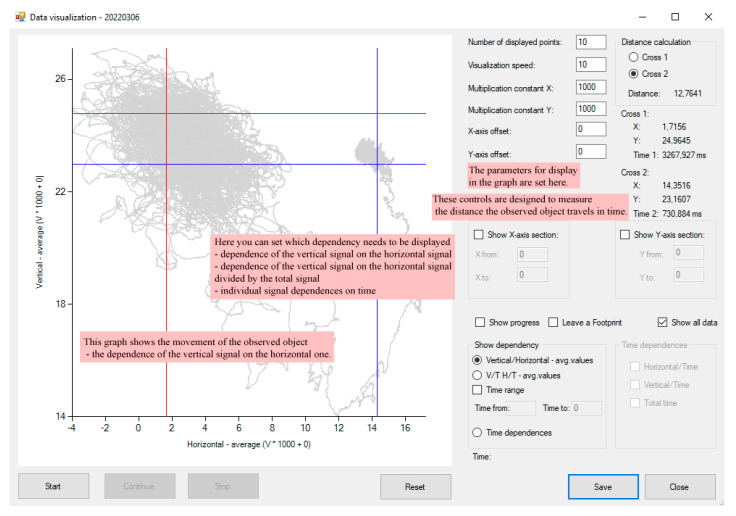
Data visualization form—vertical/horizontal dependency—the pink boxes describe each software function. The small right area presents an empty trap without a captured PS bead (noise from the background), and the larger left area shows the signal that tracks the Brownian diffusion motion particle’s trajectory.

**Figure 9:**
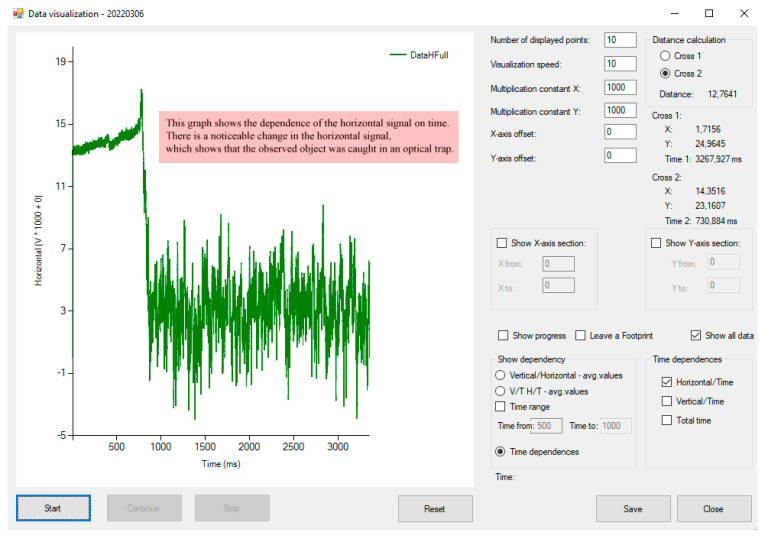
Data visualization form—time dependencies—the pink boxes describe each software function. This form supports many types of data visualization, calibration of visualized data, measure the distances between two points (cross). All specifications are set in the right panel.

In the data visualization part of the program, it is possible to import a file with measured and filtered values scanned from the detector. Imported data can be displayed in various charts and dynamically visualized to track the particle in real time. It is possible to set which signal we want to display (i.e., the dependence of the filtered values of the signal in the vertical and horizontal direction—particle’s trajectory or the time dependencies of the horizontal, vertical, or aggregated signal) (Figure 8 and Figure 9).

The evaluated data can be displayed in several ways, either statically, i.e., all the trajectory in one plot (check *Show all data*), or dynamically, when the motion of the trapped bead can be visualized (check *Show progress*) as a part of the trajectory continuously moving in the 2D space with an additional option of leaving a track of its reptation motion (check *Leave a Footprint*). The display of selected dependencies can be started with the *Start* button. The animation can be paused or stopped at any time using the *Pause* or *Stop* buttons.

The chart control, a part of Visual Studio 2012, was initially used to display graphical signal dependencies. Rendering a large amount of data took several tens of seconds, so a custom control was developed for displaying graphical dependencies. This custom control was programmed based on the creation of a bitmap image in the computer memory, which is then displayed on the form. Thus, displaying large data files takes only a few seconds.

### 2.4. Viscosity of Complex Environment

Determination of the optical trap’s force constant involves knowledge of the environment’s viscosity. In the case of a solvent consisting of a pure chemical compound, the viscosity depends only on temperature, while for the mixed solvents consisting of two miscible liquids, it depends also on the composition of the solvent. None of these dependencies are linear, therefore, a more advanced model has to be introduced to determine the viscosity of a mixed solvent for a given temperature and given composition. For a given mixture of solvents, experimental values of viscosities for a set of compositions and temperatures were found in the literature. A linear approximation of viscosity was assigned to every value from this set, which was determined as an average of the viscosities of pure components at the given temperature weighted by the molar fraction

(9) $$\eta_{lin}\left(x_{B,\;i},\;T\right)=\eta_{A}\left(T\right)\left(1-x_{B,i}\right)+\eta_{B}\left(T\right)x_{B,\;i},$$

where $\eta_{A}\left(T\right)$ and $\eta_{B}\left(T\right)$ are the dynamic viscosities of the pure compounds A and B, and $x_{B,\;i}$ is the molar fraction of the component B. The index i indicates the individual experimental points of the molar fraction for a given temperature T. Then the excess viscosity was calculated at every measured point as the difference of the experimental viscosity $\eta_{exp}$ and the linear viscosity

(10) $$\eta^{E}\left(x_{B,\;i},\;T\right)=\eta_{exp}\left(x_{B,\;i},\;T\right)-\eta_{lin}\left(x_{B,\;i},\;T\right).$$

The excess viscosity is smooth enough, therefore, it can be fitted by a polynomial of an order of 3–6 depending on the given mixture, providing a continuous smooth function $\eta_{fit}^{E}\left(x_{B};T\right)$ of $x_{B}$ with a parameter T. To determine the value of viscosity for a selected temperature within the range covered by the experimental values (but not directly in the experimental temperature values), we made use of the linear dependence of the logarithm of viscosity ln⁡η of a given liquid on 1/T, i.e., a simplified version of the Vogel–Fulcher–Tammann (VFT) equation [28]. This approximation is sufficient when the temperature range is not very wide and was proved to be valid for all the tested mixtures. Therefore, for a given $x_{B}$, a series of $\eta_{fit}^{E}\left(x_{B};T_{j}\right)$ was calculated where $T_{j}$ are the experimental temperatures. Linear regression of this series was calculated, and the obtained equation was used to calculate $\ln\eta^{E}\left(x_{B},\;T\right)$, and subsequently $\eta^{E}\left(x_{B},\;T\right)$, for any temperature from the relevant interval. Finally, the viscosity of the mixture for a given point $\left[x_{B},T\right]$ was calculated as

(11) $$\eta \left(x_{B},T\right)=\eta_{lin}\left(x_{B},T\right)+\eta^{E}\left(x_{B},T\right).$$

### 2.5. Calibrations of the Optical Tweezers

Mathematical signal analysis was used for the position and force calibration of the optical trap. The position calibration converts the detector’s data in the mV unit to the real movement of the captured particle in the nanometric unit, and the force calibration provides the trap stiffness (force constant). The calibration of the trap was tested on five measurements of spherical microscopic beads of 3.0 µm diameter (different bead in each measure) in water inside the microfluidic chip. Use of the microfluidic channel eliminates the drag flow around the captured particle.

#### 2.5.1. Trap Stiffness Determination Using Fast Fourier Transform Analysis

The force constant of a given optical trap was determined by the analysis of its force spectrum, i.e., the detected trajectory of a trapped bead transformed to the frequency domain using the fast Fourier transform (FFT) method.

As our system is based on a cost-effective diode laser, the trajectory is typically not perfectly centrosymmetric but contains some eccentricity; therefore, it has an ellipse rather than circular shape. As the orientation of this ellipse may change when the optical components are aligned, the mutual orientation of the trajectory ellipse and the image coordinate system may change uncontrollably, causing some disorder in the determined values of the force constants in X and Y coordinates. Therefore, we introduced a re-orientation of the coordinate system through the principal coordinate analysis (PCA) [29]. In case of this 2D problem its application is very simple, and the direct solution of the characteristic equation is the diagonalization of the 2 × 2 covariance matrix. This way, the coordinate system is rotated in the X–Y plane so that one axis of the coordinate system aligns with the long axis, and the other with the short axis of the trajectory ellipse. The calculation of the force constants is, thus, independent of the orientation of the ellipse.

FFT was performed using a well-established algorithm contained in the NumPy library [30,31] implemented in our program. The obtained spectrum was fitted by a Lorentz function by the method described by Berg-Sørensen et al. [13] and plotted in the log–log scale. In some cases, the Fourier-transformed trajectory (force spectrum) contained spikes, i.e., artificial peaks not corresponding with the trajectory itself but rather with frequencies originating from the vibrations of the apparatus. These spikes can be easily identified as high, narrow peaks apparently not belonging to the force spectrum. To remove them, we compare every point of the force spectrum with the moving average of a set of points around the point of interest, excluding the point of interest itself. An equal number of points are taken to the left and right side of this point (it may be chosen conveniently, typically 20 points at each side). If the difference between the point and the moving average is more significant than a chosen criterion, the value is considered as a spike and is substituted by the above-defined moving average.

The force spectrum purified from the spikes is used to determine the force constant of the optical trap. First, the frequency f corresponding to the point in which the curvature of the logarithmic plot of the Lorentzian function fitting the force spectrum is the highest (the exact method is described in [13]) is called corner frequency ($f_{c}$). The corner frequency $f_{c}$ is further transformed to the force constant κ according to the formula

(12) $$\kappa =2\pi \gamma_{0}f_{c},$$

where $\gamma_{0}$ is the friction coefficient of the trapped particle of a diameter r in an environment of the dynamic viscosity η given by the Stokes formula

(13) $$\gamma_{0}=6\pi \eta r.$$

Viscosity of the environment is calculated from its composition and temperature (described in Section 2.4).

#### 2.5.2. Position Calibration Using the Mean Square Displacement Analysis

For the correct measurements of the distances of the motion of the trapped bead, it is necessary to align the real motion of the bead (in the length units, e.g., nm) with the response of the PSD accompanying this motion. When the bead is trapped in optical tweezers generating a potential described by a quadratic function, the variance (square of the standard deviation $\sigma_{m}$) of the bead position from the trap center is [9]

(14) $${\sigma_{m}}^{2}=\frac{k_{B}T}{\kappa }.$$

Once the force constants are determined, the position calibration is achieved by the comparison of the standard deviation of the real bead position $\sigma_{m}$ calculated according to Equation (14) with the standard deviation of the detector response $\sigma_{PSD}$ calculated directly from the recorded and filtered trajectory. The deviation $\sigma_{PSD}$ is expressed either in the units of voltage (V), in the case that only the directly measured intensity difference signals are used, or as a dimensionless quantity, in the case that these signals are divided by the signal related to the total intensity registered by PSD. The position calibration constant δ is calculated as

(15) $$\delta =\frac{\sigma_{m}}{\sigma_{PSD}}.$$

The resulting δ is dependent on the averaging window used for the sliding average filtering. This window was chosen so that the filtering removes the electronics noise but keeps the details of the trajectory. It can be determined as the point in which the dependence of log⁡δ on the logarithm of the number of averaging window points has the biggest curvature.

Another method of position calibration is based on the hydrodynamic force acting on the trapped bead. When the bead is trapped, the microscope stage is moved in a selected direction and the action is recorded as a video of the microscope image. Simultaneously, the trajectory of the bead is recorded by PSD. During the motion, the trapped particle stands on its position undergoing only a small spatial displacement inside the trap enforced by the hydrodynamic friction force. Using individual frames of the video this displacement can be measured in the length units (nm). Together with this, the PSD response to this displacement is measured from the simultaneously recorded trajectory (in V or dimensionless, as at the previous method). The calibration constant δ is then determined as a ratio of these two distances.

#### 2.5.3. Construction of Bead Position Distribution Function

Furthermore, the distribution function of the bead position in the trap can be constructed, and the potential function of the trap can be found using the Boltzmann distribution of energies. The distribution function, i.e., the frequency of occurrence of the bead on the coordinate x, is given by

(16) $$P\left(x\right)=\frac{\exp\left(-\frac{V\left(x\right)}{k_{B}T}\right)}{\int_{-\infty }^{\infty }\exp\left(-\frac{V\left(x\right)}{k_{B}T}\right)}=\frac{\exp\left(-\frac{V\left(x\right)}{k_{B}T}\right)}{Q},$$

where $V\left(x\right)$ is the trap potential, and Q is a partition function. Hence, the ratio of these frequencies for a point x and the potential minimum $x_{min}$ is

(17) $$\frac{P\left(x\right)}{P\left(x_{min}\right)}=\exp\left(-\frac{V\left(x\right)-V(x_{min})}{k_{B}T}\right).$$

Setting $V\left(x_{min}\right)=0,$ we obtain $\frac{P\left(x\right)}{P\left(x_{min}\right)}=\exp\left(-\frac{V\left(x\right)}{k_{B}T}\right)$, and the potential of the trap $V\left(x\right)$ can, thus, be calculated using the formula

(18) $$V\left(x\right)=-k_{B}T\ln\frac{P\left(x\right)}{P\left(x_{min}\right)}.$$

## 3. Results and Discussion

The optical trap data evaluator program is designed to capture and evaluate data when working with an optical trap. The program allows the operator to adjust the line of the incident laser beam by the movable mirrors and to set the detector to acquire the light emitted by the sample. Furthermore, this software enables measurement management with the possibility of storing all measured data and their evaluation.

### 3.1. Force Constant of the Trap

Force constants of the optical tweezers apparatus were determined by the commonly used method of analysis of the power spectrum of the trajectory of trapped 3 µm and 0.8 µm (in diameter) PS beads. This analysis is quite straightforward and is carried out for every trapped bead before the subsequent measurement.

**Figure 10:**
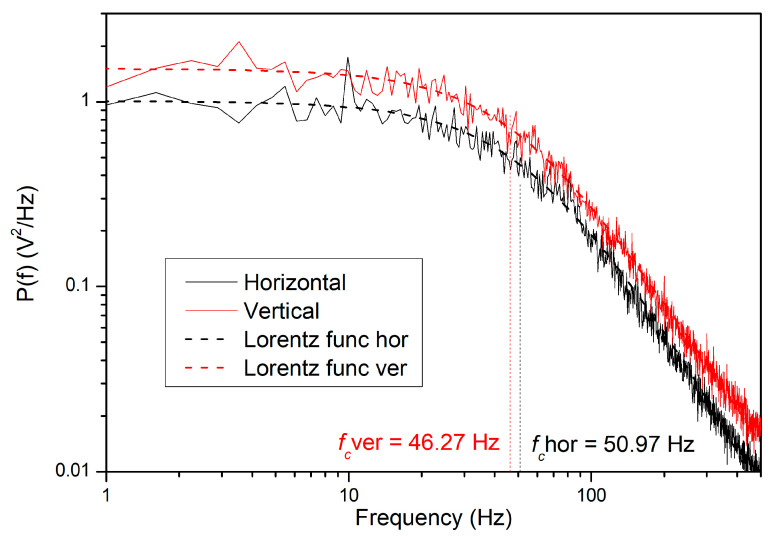
Fast Fourier transform (FFT) of the filtered signal of 3.0 µm PS bead (solid lines) with Lorentz function fit (dashed lines) showing the point of maximum curvature of this function $f_{c}$ divided on two perpendicular axes: horizontal (black) and vertical (red).

In the example shown in Figure 10, the trajectory of the 3.0 µm PS bead was acquired for 100 s with a scanning frequency of 100 kHz and filtered using the blocking average over 10 points. The filtered signal was subsequently transferred using FFT and expressed in units of V^2^/Hz.

The high-frequency parts of the two curves should be the same in calibrated OT for both directions. However, the curves are taken as noise-eliminated raw data from the PSD detector. As we measure the light of the trapping beam reflected by the bead, the absolute value of the detector signal is dependent on the intensity of the light focused to the bead. As the beam of the used laser is not perfectly cylindrically symmetric, the illumination of the bead in the two directions is different, therefore, the absolute values of the signals differ slightly as well.

Based on the knowledge of $f_{c}$ the trap stiffness κ can be calculated using Equation (12)

(19) $$\begin{array}{c} \kappa_{ver}=2\pi \gamma_{0}f_{c}=12\pi \eta rf_{c,ver}\\ =12\pi^{2}\times 0.910\times 10^{-3}\;Pas\times 1.5\times 10^{-6}\;m\times 46.27\;Hz\\ =7.48\times 10^{-6}\;\frac{N}{m}. \end{array}$$

An average of five different measurements results in $\kappa_{ver}=7.03\pm 0.15\;\frac{pN}{µm}$. Due to the small anisotropy of the generated trap, the force constant was also calculated in the second axis separately, which results in $\kappa_{hor}=8.13\pm 0.09\;\frac{pN}{µm}$.

### 3.2. Filtering the Trajectory Signal

**Figure 11:**
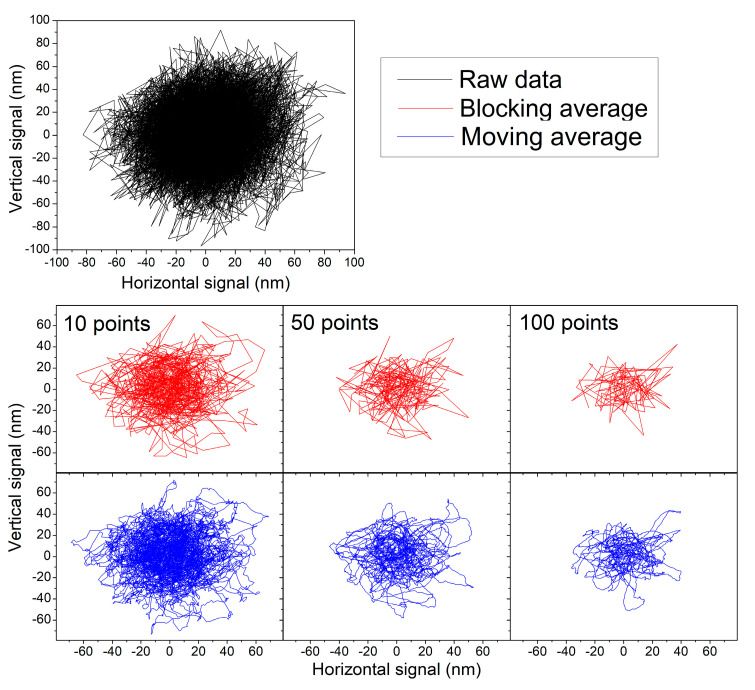
Influence of blocking (red line) and moving (blue line) average on measured and calibrated raw data (black line) of 3 µm PS bead trajectory. Raw data were measured 100 s with 100 kHz sampling frequency reduced over 10 points by blocking average and only first second was visualized. The trajectory is filtered using 10 (left column), 50 (middle column), and 100 (right column) average windows. All axis in six panels are shown in the same scale demonstrating the signal compression.

Various applications of optical trap require the precise measurement of the trajectories of the trapped objects. Therefore, the primary position signal produced by the moving trapped object must be recorded and filtered from the noise caused by the electronics. We tested the influence of data filtering on the shape of particle’s trajectory (Figure 11).

**Figure 12:**
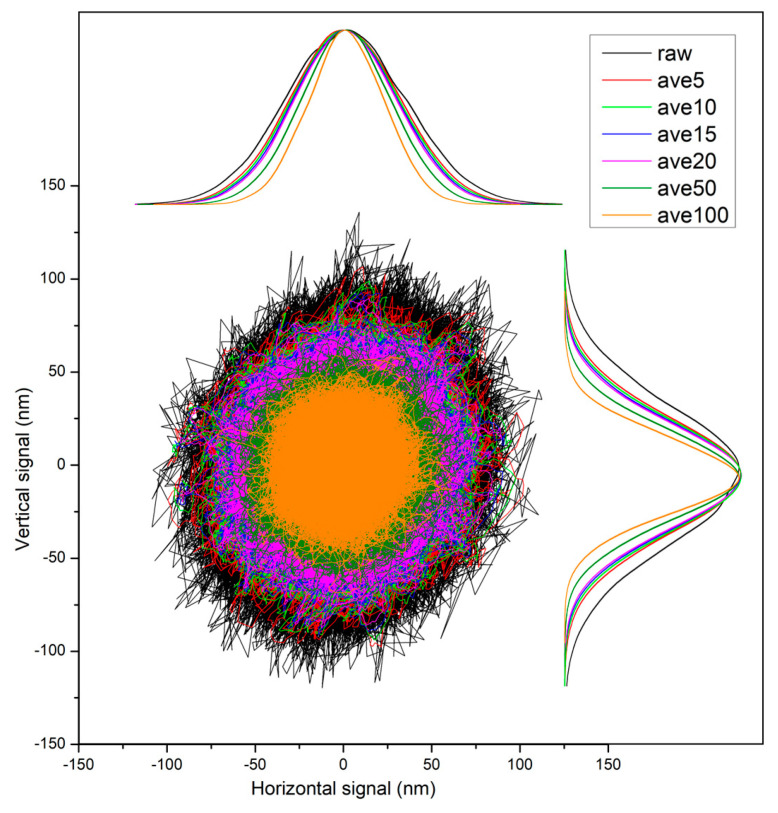
Comparison of the noise reduction using moving average filter on calibrated signal of 3 µm PS bead trajectory. Raw signal (black) contains the electronical noise (see sharp spikes). Other colors present the same calibrated trajectory moving averaged from 5 to 100 points. All curves are normalized to equal amplitude.

Moving averaging data over 10 points presents clear and detailed particle trajectories (Figure 11) without noise and deformations by the mathematical operations. Comparison with the raw signal indicates that the majority of the noise was removed, and the trajectory shows quite tiny details of its shape. When more points are taken to the averaging window, these details are being wiped and the trajectory bundle shrinks, resulting in an artificially simplified trajectory shape. A more detailed view can be seen in Figure 12. It shows that averaging over 5 points still contains some noise (sharp discontinuous spikes), but over 10 points, the trajectory starts to be smooth. Averaging from 10 to 20 points is relatively stable (see distribution functions), but when a bigger window (50 points) was used, the trajectory started to be compromised and deformed by flattening of its genuine short-time features. Thus, averaging over more than 20 points seems to be undesirable. The acquisition rate of all moving averaged trajectories is 10 samples/ms, given by the original 100 kHz sampling frequency and the initial blocking averaging over 10 points. This rate is sufficient to record the trajectory with enough details.

When the blocking average is used (Figure 11—red curves), the shrinkage of the trajectory bundle with the increasing length of the averaging window is similar to that of the moving average. However, the trajectory is not smooth as the number of trajectory points decreases proportionally to the window length. The trajectory, thus, loses the tiny shape details. Therefore, the blocking average was not used as the method of noise removal from the trajectories. 

**Figure 13:**
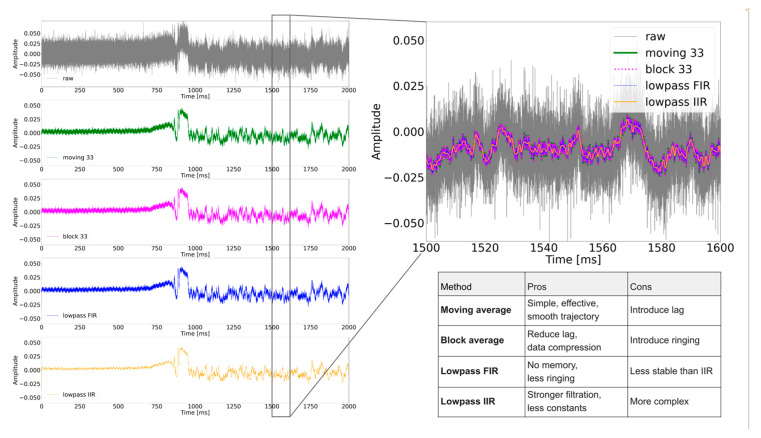
Comparison of the noise elimination methods. Before the signal changed, there was an empty trap without captured PS bead (noise from the background) and when the signal changed, the PS bead fell into the trap, and the signal tracked the particle’s diffusion motion. Raw signal (grey line) is acquired with 333 kHz sampling frequency and filtered over 33 points by moving average (green line) and blocking (purple line). Cutoff of FIR (blue line), and IIR (yellow line) was 1 kHz, and order of the FIR filter was 33. The IIR factor was set to 3. The signal detail is shown for 100 ms, and under that is the table with summary of advantages or disadvantages of used methods.

In addition to the use of moving average filter for purifying the trajectory from the noise, different filtering methods (described in Section 2.2) were used. Their performance was compared on the measured data of the captured 3 µm PS bead (Figure 13). This comparison indicates that all the methods provide a sufficient level of noise removal and, thus, a good quality of the filtered signal. Therefore, we use the simplest method of moving average for the further work with the trajectory. However, a slightly higher purity of the signal was reached using the lowpass IIR filter that provides a relatively smooth curve while still keeping sufficient detail of its shape. Further investigation of this filter in the context of our experiments is planned for the future. 

This comparison between the empty trap (Figure 13 from 0 to approximately 700 ms) and the trapped bead (times from 1000 ms higher) shows the elimination of noise to approximately zero amplitude in an empty trap. On the other hand, the bead trajectory with the low-frequency motion still tracks the bead position after filtering (higher amplitude). For tracking the bead position, the optimal filter is the moving average as a type of low-pass filter in the context of the used average window [15] regarding the reasonable sampling frequency of these types of tracking objects.

### 3.3. Position Calibration of the Trap

To measure the distances of the motions of the trapped objects, it is necessary to also calibrate the distance in the X–Y plane (perpendicular to the optical axis) in which the motion of the trapped bead takes place, i.e., to align the signal on the detector output (signals in the horizontal or vertical axis divided by aggregated signal—in arbitrary units) with the distance traveled by the trapped bead under microscope (length, in nm). Two methods can be used for this purpose. The first one, noted as σ-method, compares the variance $\sigma_{m}$ expressed from Equation (14) with the variance of the PSD signal $\sigma_{PSD}$ produced by the trapped bead. The computed $\sigma_{V}$ from Gauss fit of the five different 3.0 µm PS bead trajectory position distributions in voltage was $\sigma_{V,ver}=2.80\;\pm \;0.08mV$. This was subsequently recalculated to nm, and $\sigma_{m}$ calculated using Equation (14) with the same example as used in Section 3.1 is

(20) $$\sigma_{m,ver}=\sqrt{\frac{k_{B}T}{\kappa_{ver}}}=\sqrt{\frac{1.38\times 10^{-23}\;\frac{J}{K}\times 297.25\;K}{7.48\times 10^{-6}\;\frac{N}{m}}}=2.34\times 10^{-8}\;m.$$

Hence, the ratio of the computed $\sigma_{V}$ corresponds to the motion of the trapped bead $\sigma_{m,ver}=24.19\pm 0.27\;nm$ (in Equations (19) and (20) only one example from five is present) using Equation (15), resulting in the calibration constant $\delta_{ver}=8.68\pm 0.34\;nm\cdot mV^{-1}$, indicating a good sensitivity for measuring this kind of trapped object with the PSD detector. This result demonstrates the detector’s calibration constant in the vertical axis. The calibration constant result for the horizontal axis using the same procedure is $\delta_{hor}=6.01\pm 0.11\;nm\cdot mV^{-1}$.

**Figure 14:**
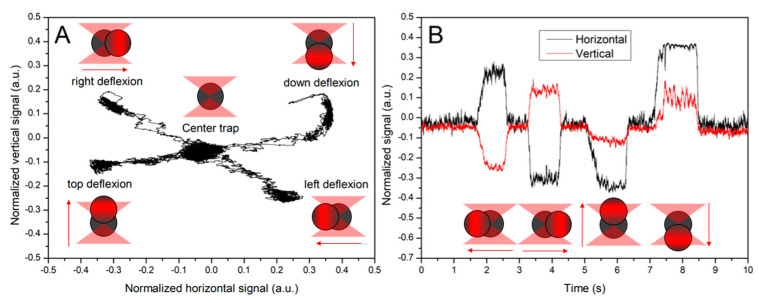
Detector normalized signals moving averaged over 50 points from 3 µm PS bead deflection outside the trap’s center. Panel (**A**) shows the bead trajectory in four perpendicular directions, with markers illustrating the direction of the bead shift. Panel (**B**) shows the time dependency of horizontal (black line) and vertical (red line) signals.

The second hydrodynamic method compares the signal change with the shift observed directly on the screen, visualizing the microscopic image when the bead is shifted in the trap by a known hydrodynamic force. This method can be considered less precise and more cumbersome to carry out but more reliable, as the shift of the real position can be seen directly on the display, giving us a certain confidence that we do not measure artifacts. The results presented in the normalized form of both signals are shown in Figure 14 and Table 1. 

**Figure 15:**
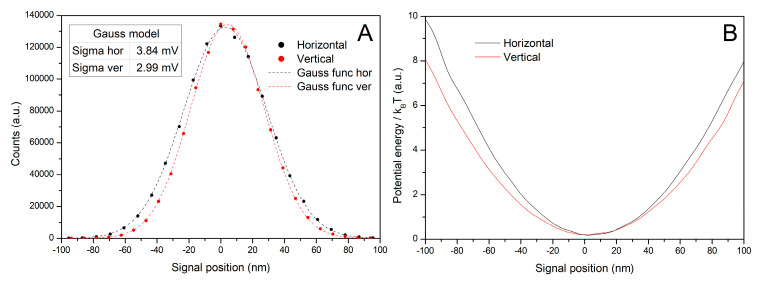
Distribution of a trapped bead position (**A**) and optical trap potential energy in k_B_T units (**B**) of the 3.0 µm bead’s trajectory as a function of its distance from the trap center (minima of the curves are aligned to zero). Dots in panel (**A**) present the frequency of the bead occurrence fitted by Gauss function (dashed lines) in horizontal axis (black) and vertical axis (red) in varying distances recalculated to the real movement of the particle in nm scale from the trap center.

Distribution function of trapped PS bead (Figure 15A) and finally, the potential of our generated trap (calculated using Equation (18), Figure 15B) given in units of $k_{B}T$, was calculated.

**Figure 16:**
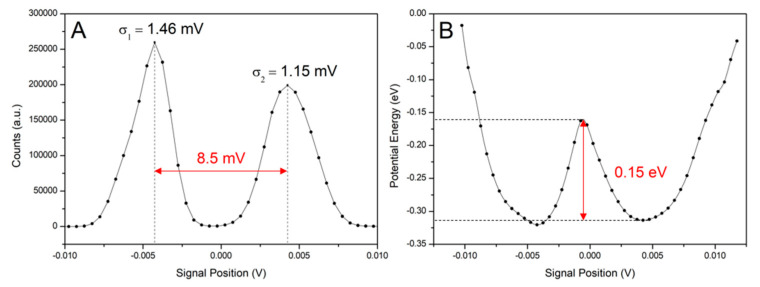
Position distribution of the bead’s trajectory (**A**) and potential of two optical trap centers (**B**) for 0.8 µm PS bead in vertical axis. The scanning frequency was 333 kHz, averaged over 33 points by blocking average.

A drawback of the use of the diode laser is that the beam does not originate from a small circular spot but from a line of the emitting centers. Therefore, the beam does not behave ideally and may produce some artifacts. In some cases, the formation of a series of optical traps located relatively close to one another (typically several µm) can occur, especially when smaller beads are trapped, e.g., beads of the diameter of 0.8 µm instead of 3.0 µm. Both the traps are strong enough to protect the bead from hopping between them, but the jump from one to the other can be induced by a sudden motion with the stage or tapping it by hand. After recording the signal from both traps, the distribution function of the bead (Figure 16A) occurrence, as well as the potential of the trap (Figure 16B) can be evaluated. The force and position calibration of the trap results in $\kappa_{1}=5.03\cdot 10^{-6}\;\frac{N}{m}$ and $\delta_{1}=19.8\;nm\cdot mV^{-1}$ (from the calculations presented higher) for the left peak and $\kappa_{2}=3.05\cdot 10^{-6}\;\frac{N}{m}$ and $\delta_{2}=32.2\;nm\cdot mV^{-1}$ for the right peak. The distance between the traps can be easily measured from the microscope image—in our experiment, the distance is 2.04 µm. Considering that the widths are in the order of lower tens of nm, the position calibration is not applicable to the long distance between the traps, although it gives reliable results inside each trap. The reason is the inconsistency in the illumination of the bead between the two traps, resulting in the fact that the reflected light from the region between the traps is not comparable to that in them.

The potential of our generated trap (Figure 16B) with both centers separated by approximately 0.15 eV energy barrier was also calculated using Equation (18). Thus, even in this case, both traps can be used for the force measurement, provided that the trapped bead stays in one of them and does not jump between them.

### 3.4. Trapping Properties of the Constructed OT in Comparison with Other Devices

The optical trap device is based on the diode laser that does not provide such a high-quality laser beam as other lasers used for this purpose. The emitting center is not a small spot but rather a short line, therefore, the rays of the beam cannot reach perfect parallelism by using standard lenses in the optical path. Consequently, the focusing of the beam to the trapping point is less precise. Moreover, the cross section of the beam intensity is not radially symmetric but possesses a slight eccentricity. These phenomena decrease the force constant of the optical trap and cause differences in the trapping constants in different directions in the focal plane. In spite of that, the device shows a good quality for the purpose of single-molecule experiments [3,32,33], study of the complex mixed solvents [34,35], or thermophoretic measurements [36,37,38]. The force constant obtained with the 3 µm PS beads is about 7–8 pN/µm. This is on the lower end of the range of the force constants reached on different devices [3,7,11,39,40,41,42], but in some cases even smaller values were used [36,43]. Our device can, thus, be used for exerting the forces in the range of units, at most low tens, of pN/µm. These are the typical forces for, e.g., kinesin [44,45,46], myosin [47,48], and dynein [49] molecular motors. Phenomena like protein unfolding or separation of DNA strands usually require slightly higher forces, but even in this field some phenomena are observable in the range that can be covered by our device [32,50]. Some non-biological studies used the traps of force constants similar to ours, e.g., trapping the organic microdroplets in water [34] or thermophoretic experiments [36].

The position calibration constants of our trap are about 1000 nm/a.u. (the relative unit given by the ratio of the difference signal and the total signal). As the detector is able to reproducibly detect the differences in the order of 0.001 a.u., the sensitivity of the position detection is in the nanometer order, which is sufficient for all kinds of experiments cited in the previous paragraph. 

Our apparatus is, thus, comparable with other devices reported in the literature, although it is not applicable to experiments in which high force constants of the traps are needed. Further improvement of the trapping properties of the device will be the matter of continuation of this research, especially improvement in the optical properties of the beam, in addition to others by correcting the beam eccentricity using the cylindrical lenses. Nevertheless, there is still a wide field of applications that do not require such high forces and can be served by this device. 

## 4. Conclusions

The dual optical tweezers apparatus based on the fluorescence microscope Olympus IX-73 and a cost-effective IR trapping diode laser of 808 nm wavelength and 3200 W power was constructed in our laboratory and equipped with an integrated software package. It allows the operator to control the instrument, calibrate it, record the measured data, and carry out post-processing and visualization. All the software features were designed to be user-friendly and integrated into an intuitive user interface. The device was tested on trapping 0.8 and 3.0 µm polystyrene beads, and the force and position calibration of the traps were carried out. The test indicated good sensitivity of the instrument for the position and force measurements and proved the full functionality of the software package. It was also shown that the device is applicable in spite of using a relatively cheap IR diode laser with a non-ideal beam profile, still keeping a good position and force sensitivity. The device equipped with this software opens the way to carrying out various single-molecule experiments in the field of physical and biophysical chemistry.

## Figures and Tables

**Table 1:** Evaluation of calibration constants in both perpendicular directions by two different methods.

Hydrodynamic Method				σ-Method	
Direction	Deflection		Calib. Const. (nm/(a.u.))	Detector Variance $\sigma_{PSD}$ (a.u.)	
	Detector (a.u.)	Camera (nm)		Horizontal	0.024
Left	0.363	380	1046.54	Vertical	0.015
Right	0.368	330	897.71	Calculated Variance $\sigma_{m}$ (nm)	
Down	0.417	620	1487.88	Horizontal	23.475
Top	0.333	510	1530.15	Vertical	26.823
$\delta_{hor}$ ^a^ (nm/(a.u.))			972	$\delta_{hor}$ (nm/(a.u.))	983 ± 66
$\delta_{ver}$ ^b^ (nm/(a.u.))			1509	$\delta_{ver}$ (nm/(a.u.))	1804 ± 59

^a^ Average value of left and right directions. ^b^ Average value of down and top directions.

## Data Availability

Data are contained within the article.
